# Students perspectives of the effect of ethnicity on experiences in a graduate entry medicine course in Wales: a qualitative study

**DOI:** 10.1186/s12909-023-04852-7

**Published:** 2026-02-17

**Authors:** Mehwaish H. Zulfiqar, Mariam Moughal, Arian Rahim, Jamie Tomlin, Kimberly Tin, Funminiyi Obilanade, Lydia Brown, Kamila Hawthorne

**Affiliations:** Swansea University, Swansea, UK

**Keywords:** Black, Asian and Minority Ethnic (BAME), Graduate Entry Medicine (GEM), General Medical Council (GMC)

## Abstract

**Objectives:**

To explore the clinical experiences of graduate-entry medical students; whether ethnicity impacts this and how medical education can better prepare students from a diverse range of backgrounds.

**Design:**

Qualitative study using semi-structured interviews.

**Setting:**

A medical school in Wales.

**Participants:**

Seventeen graduate-entry MBChB students were recruited using volunteer and snowball sampling; 9 students self-identified as being from Black, Asian or Minority Ethnic backgrounds (BAME) and 8 from White Caucasian backgrounds.

**Results:**

When asked whether they felt they had experienced disadvantage during their time on the course, 6 BAME students report feeling disadvantaged during their studies and 2 were unsure. This was compared to 2 white students who felt disadvantaged. Gender was most frequently linked to disadvantage, followed by ethnicity and racial background. Patient interactions were most linked to microaggressions and overt racism, leading to uncomfortable situations for BAME and White students. Clinician interactions were identified as a source of disadvantage, often linked to students’ being overlooked in teaching and opportunities. ‘Unfamiliar’ names were associated with negative experiences, which ranged from being blanked for having a name perceived as difficult to pronounce to being disrespected.

Microaggressions by clinicians and patients were identified by BAME and White participants alike, with participants feeling unsure of how to handle them. Institutional factors were divided into language used by lecturers and clinicians and lack of support. Language was felt to be out-dated and furthering the feeling of ‘other’ felt by BAME students. Students reported feeling unsupported and dismissed when trying to escalate issues, leading to a lack of trying after a while.

**Conclusion:**

In this cohort, although patients were repeatedly linked to discrimination, the disadvantage in medical education was perceived to be impacted most by clinician interactions. Names and being ignored by clinicians most impacted on learning experiences. Institutional factors compounded this and reinforced the feeling of ‘other’ by BAME students.

**Supplementary Information:**

The online version contains supplementary material available at 10.1186/s12909-023-04852-7.

## Background

The UK’s General Medical Council (GMC) states that medical education should be fair, based on principles of equality and diversity and provide a supportive educational environment to ensure students’ learning potential is achieved [1]. Across United Kingdom (UK) universities, it has been recognised that students from ethnic minorities have differential attainment when compared with their white peers in written and clinical examinations during university and then continuing into postgraduate training [2]. Approximately 39% of the 7500 medical students in the UK are from a Black, Asian or Minority Ethnic (BAME) background [3] which makes this an important consideration for medical education. After correcting for factors commonly thought to contribute to differential attainment, such as socioeconomic status, language proficiency and other medical student characteristics, there remains a difference in medical school finals outcomes [4]. It is important to identify and investigate factors during medical training which could contribute to this differential attainment and ways to reduce it.

Previous studies in medical schools in England have found that ethnic minority healthcare students have higher stress compared to their white peers and qualitative interviews with trainee doctors showed strained relationships with seniors due to a perceived bias during work-based assessments and the psychological impact of stereotypes [5, 6]. This study describes the experience of BAME medical students in Wales, which has a less ethnically diverse local population (93.8% “White” ethnic groups) than the rest of the UK (81.7% “White” ethnic groups across England and Wales) [7]. This is in addition to students being on a Graduate-Entry Medicine (GEM) course, which tends to have significantly lower proportions of BAME entrants compared to undergraduate medical courses [8]. This heightened minority gradient means that this group of students are at risk of being further marginalised. The study was conducted in 2021 and largely refers to students’ experiences prior to the events of 2020, when the COVID-19 pandemic brought to light inequalities the way in which healthcare inequalities led to a significant proportion of deaths among BAME populations. This study was undertaken as part of a student-selected project as part of the Developing Professional Practice module in the Swansea GEM course. The study examines the clinical experiences of these students to identify areas where BAME students feel adversely impacted.

## Aims

1. Does ethnicity impact medical students' experiences in clinical settings?
2. Are other protected characteristics implicated (sex, age, disability, gender reassignment, marriage and civil partnership, pregnancy and maternity, religion or belief, sexual orientation)?
3. How can medical education prepare students and staff to tackle discrimination in clinical settings?

## Methods

### Design

This study used a qualitative 1:1 semi-structured approach to explore medical students’ experiences and perceptions of factors underlying this. Interviews were conducted online with the lead author from January 2021 to February 2021. Questions explored experiences in clinical settings, perception of disadvantage, views on discrimination in healthcare settings and improvements to preparation for clinical placements. The principal researcher conducting the interviews was a graduate-entry medicine student and of a South-Asian background (MZ). An encrypted audio recorder and transcriber was used for data capture.

### Sampling strategy and recruitment

Invitation to interview was extended to all students in the four cohorts of the MBChB course at the medical school, which amounts to approximately 400 students. Both BAME and White participants were included in the sampling frame. BAME participants included those who identified as being from African, Asian, Arab or Caribbean descent.

Participants were recruited through direct contact with the researcher, email invitation and social media recruitment through the medical school’s BAME student network. All students who expressed an interest in the study were provided with a participant information leaflet and a consent form. Twenty students expressed an interest - ten from BAME backgrounds and ten from White backgrounds. Due to availability, nine interviews with participants from BAME backgrounds and eight from White backgrounds were organised in January and February 2021. Interviews were conducted on Zoom in private rooms for both the participant and interviewer. Interviews ranged from approximately 10 minutes to 45 minutes, with an average length of 24 minutes.

### Data collection, processing and analysis

Due to the COVID-19 pandemic, all interviews were conducted via Zoom, a video conferencing platform, by the principal researcher MZ - all interviews were kept confidential and carried out in a private space. Prior to each one, participant consent forms were obtained by MZ, in addition to verbal consent being sought out. Interviews were audio recorded, transcribed verbatim, and anonymised. Once transcribed and analysed, codes were generated from the data, using a reflexive thematic analysis approach. Recurring patterns of codes and codes generated by data reviewed by KH (academic supervisor to the lead author), were then used to generate the themes.

## Results

### Participants

Seventeen participants took part in the study. Demographics were self-reported by students as part of the questions asked (see Additional file 1). Demographics are as follows: nine BAME students, eight White students. Out of the BAME students who partook, five were Asian and four were Black/Mixed. Twelve of the participants were female, seven of whom were BAME students, and five who were White students. The majority of participants were in their clinical years of study - thirteen students were in their third year, three in fourth year, and one in second year.

### Disadvantages

**Fig. 1:**
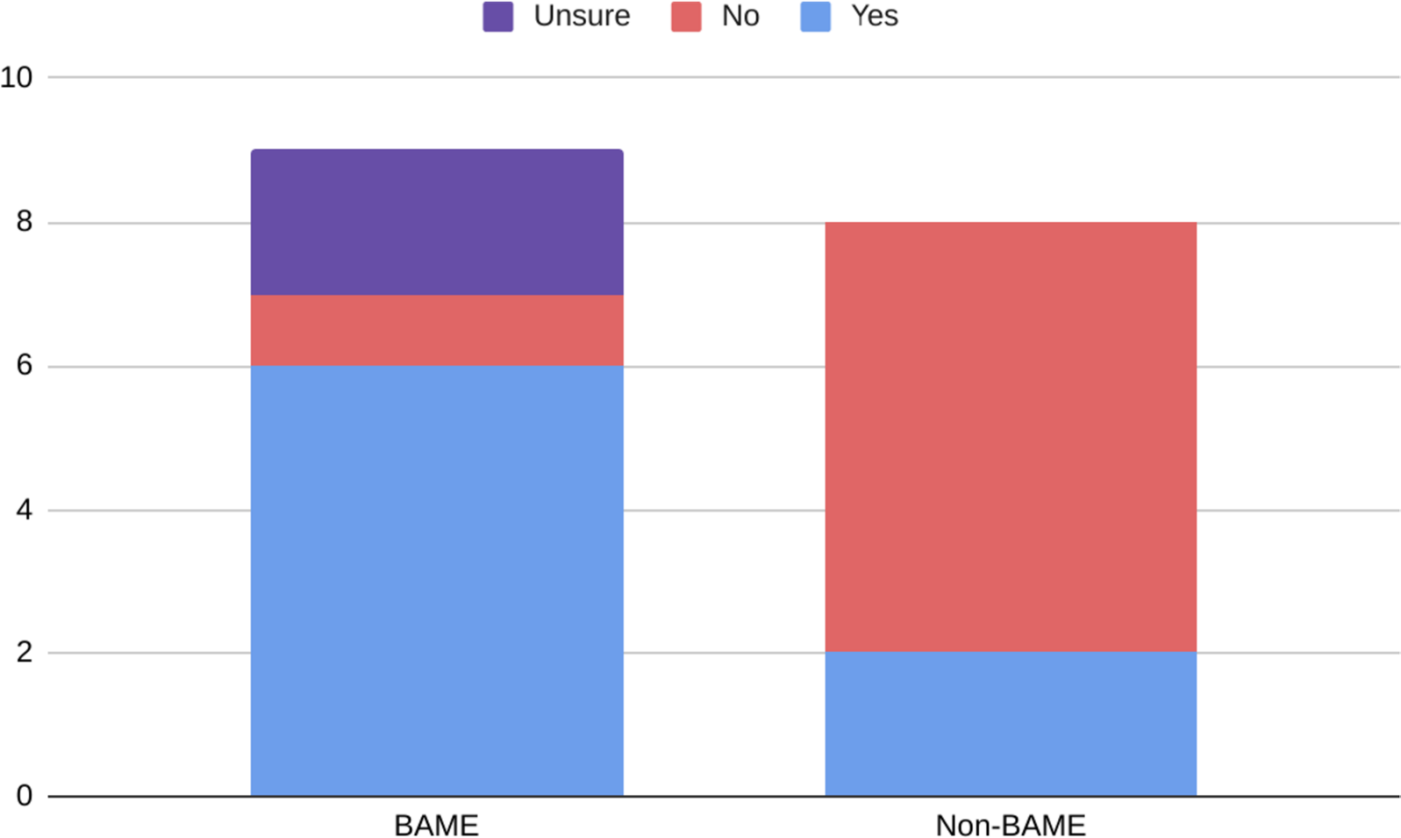
BAME and White participants’ response to the question “during your time studying medicine, have you ever felt like you have been at a disadvantage or given less opportunities?”, categorised into “Yes” (blue), “No” (red) or “Unsure” (purple) categories

When asked “during your time studying medicine, have you ever felt like you have been at a disadvantage or given less opportunities?” six BAME participants compared to two non BAME-participants said yes. Out of all participants who responded no, only one was of a BAME-background, compared to six from White backgrounds (Fig. 1).

From the BAME participants who affirmed the question, the highest incidence of disadvantage was reported by Black/Mixed participants-three (75%) responded yes, whilst one (25%) stated that they were unsure. Moreover, three (60%) Asian students also confirmed that they had felt as though they had been at a disadvantage, whilst one (20%) reported that they hadn’t, and one (20%) stated that they were unsure.

Notably, eight (61.5%) female participants reported that they were at a disadvantage, whilst none of the male participants reported a disadvantage.

**Fig. 2:**
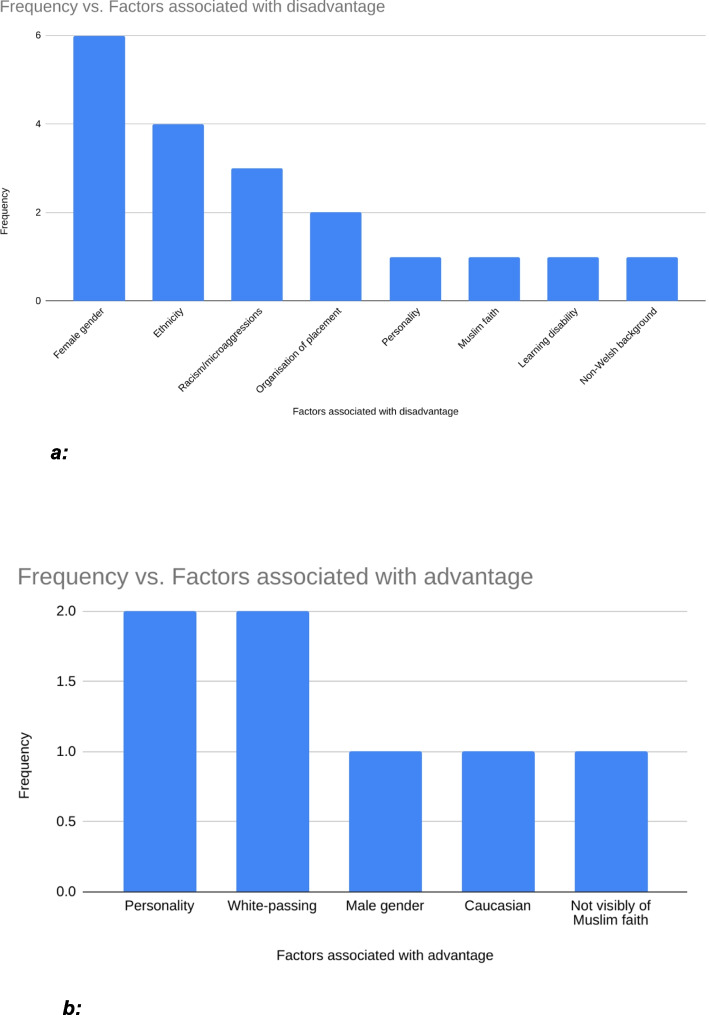
**a** Factors associated with disadvantage vs frequency of being mentioned by participants. **b** Factors associated with advantage vs frequency of being mentioned by participants

Incidentally, female gender was highlighted as the most prominent factor which contributed to disadvantage, with a frequency of six (Fig. 2a). Ethnicity was reported as a contributing factor four times, racism and microaggressions three times and placement organisation was mentioned twice. Factors reported once each include personality, learning disability,

Muslim-faith and belonging to a non-Welsh background (Fig. 2a). Conversely, advantageous/protective factors identified twice included personality and being White-passing (Fig. 2b); male sex, being of a White-background and “not being visibly Muslim” were reported once each as being protective factors. Protective factors were factors felt to be causes for BAME or non-BAME participants for them to not receive discrimination or even put them at an advantage.

### Themes identified

Three main causal themes were identified during this study - clinician interactions, patient interactions and institutional factors. Clinician interactions were further broken down into being ignored, name mispronunciation and avoidance, and microaggressions. Institutional factors were divided into language and support. Due to the conversational nature of the study, these themes overlap and interact.

### Clinician interactions

#### Ignoring

Being ignored on placement was widely reported from all participants, however this was found to be more frequent for BAME and female students. When partnered with a ‘white male’ student, several participants noted that being ignored by clinicians was more apparent, as evidenced through lack of eye contact and rarely addressing the BAME or female student. One BAME student highlighted this lack of recognition by describing how they had to be louder to gain equal opportunities.

*“There’s a natural gravitation especially towards white males, who come across as a bit more, not dominating, but maybe a bit more direct. And so they get more opportunities. I do think that when you're in what's considered a minority, you have to kind of be a bit louder to be given equal opportunity.”*

*-Female, Asian*

Another participant from a BAME-background reported that in certain environments they found that their race became a focal point, causing them to stick out. The theme of being ignored was interlinked with gender biases. Two female participants reported how opportunities were offered to them under intimidating circumstances by male consultants, feeling that their gender made them targets for intimidation. Noticeably, surgery was highlighted in several discussions regarding gender bias and lack of opportunities. Three students reported how clinicians mistook them for nurses, contributing to differential treatment. Moreover, intersectionality was highlighted amongst several students, particularly Black females; this caused additional strain on their relationships with certain clinicians.

*“I’ve had opportunities offered to me, but only under very uncomfortable circumstances like the consultant specifically wants to pick me up from somewhere himself and drive me back home.”*

*- Female, Asian*

*“I think definitely I’d say race, but mostly I thought it was gender as well. They were saying I was a nursing student [...] so yeah so both.”*

*- Female, Black*

*“Both me and the other female student had quite a similar experience, whereas by my being with white male students I've noticed there is like discrepancy in the experience we have - just who’s kind of interacted with the most ... just like how much eye contact we’re given when we're having a teaching session and some of its like questions were asked. I think that's definitely an intersectionality.”*

*- Female, Mixed*

#### Names

Name avoidance or mispronunciation was identified as a discriminatory factor in almost all interviews. Most discussions that mentioned names, also explored the theme of being ignored. Participants highlighted how clinicians would avoid addressing names that they were unfamiliar with or unsure of how to pronounce. This, again, was associated with having to actively push to be heard by one participant, and reduced opportunities by others.

*“When people introduce themselves - say if three people say like two Welsh names or two English names and then one like Asian name or an African name and the person is just like ‘oh those two will be easy to remember’ and then immediately there's a divide [...] those two people will be remembered and maybe even asked questions directly, then the one other person will sort of blend into the into the background - and will have to actively push to be heard, so that that definitely does impact how people are treated and it's quite visible.”*

*- Female, Black*

A few students compared this to clinician’s willingness to say Welsh names, which may also be viewed as difficult to pronounce - with these students however, there was not the same avoidance. Two students reported that nursing and associated healthcare colleagues had offensively commented on their names. They linked this to a perceived reduced standard of professionalism, whereas they felt that doctors were held to a higher standard.

“A nurse rolled her eyes and was like ‘my name is so much easier than yours’, and then I said ‘I think it's easy for *region of my name origin*’ and then she understood that she upset me [...] I just would like to say that it can genuinely ruin someone's day if people don't know what your name is or they can't read it, or if they react negatively.”

*- Female, Black*

#### Microaggressions

BAME students reported numerous occasions of microaggressions and subtle discrimination, which were also picked up on by many White participants.

*“I’m sure there is definitely still a lot of biases and a lot of prejudice [...] but it’s definitely more hush hush, because as professionals you're not going to be outwardly racist or outwardly sexist homophobic, you’re gonna keep your biases yourself, because you know that that won’t fly, whereas a patient or the system can be more outwardly offensive.”*

*- Female, White*

*“I think they hide it well, because they try to be professional. I think you can tell where they'll avoid you more on placements. Rather than try and help you, they will just like leave you alone, [...] and they won’t necessarily support you, so they do it politely”.*

*- Female, Black*

BAME students noted an underlying understanding of why a clinician may not be engaging with them as much as other students, or why their experiences with certain clinicians were different to their White peers. White students observed more discriminatory comments from clinicians and colleagues and reported feeling unsettled due to being unsure of how to appropriately respond.

#### Patient Interactions

All participants attested to the question “do you think race or ethnicity play a factor in how people are treated by patients?”. Patients were found to treat doctors from ethnic minority backgrounds differently, particularly those with ‘foreign’ accents. Moreover, BAME students reported that patients were the most likely group to show racism and microaggressions; again, racist experiences from patients were most reported by Black participants.

*“There’s so many things that are racially aggressive and just racist things that will happen on placement, so I find that a lot of the time. It’s mostly the patients who say things, but because of the silence of the members of staff it just makes it like a double whammy. It's like you’ve got this patient who has been racist and they’re a patient, so you can’t say anything.”*

*- Female, Black*

Generally, BAME students identified difficulty in building a rapport with patients, and two students mentioned how patients who they had spoken to over the phone initially would change their behaviour when they realised that the student was from a BAME-background. Black students reported more overt racism, and in general participants reported intrusive patient questioning, such as “where are you from?”—this was met with an unwillingness from the patients to accept a city in the UK. White participants witnessed racism from patients, particularly to other doctors, but also generally, as patients assumed that they would agree with them.

Several of these students expressed frustration in not knowing how to respond in these situations.

*“Patients do turn around and say ‘I just can’t understand you, I don’t know what you're talking about, I don’t want to speak to this doctor’ and I’ve seen it happen multiple times to people from different ethnicities, different countries, different accents and I just find it really, really infuriating.”*

*- Female, White*

*“I’ve had patients refuse to answer pretty important questions in a history because they’re stuck on my name, and even if I'm like ‘I’m from London’. ‘Okay well, where are you really from’, and eventually it all comes down to the same comment ‘well you're not from here, because you don't look like you're from here - your hair’s too dark, your eyes are too dark, your name is not right, you’re not from here, I don't know why you're lying to me’.”*

*- Female, Asian*

### Institutional factors

#### Language in teaching

All students highlighted how they felt that their university curriculum lacked diversity. A ‘White-centric’ curriculum was accentuated, whilst other ethnicities were typically only mentioned in the context of stigmatising illnesses. Participants from both BAME and White backgrounds had witnessed lecturers using uninformed language, make generalisations about entire continents (e.g. “in Africa”), and speak about non-Western cultures in a derogatory manner.

*“That’s really one of like three things you’re taught about that specific patient population, so you just associate those kind of stigmatising illnesses with any kind of patient of that specific ethnicity that comes in, like Asian people are much more likely to get diabetes, without any regards for the larger health makeup [...] White is very much seen as a baseline, which is understandable in the UK, but the specific context to a lot of that teaching is lacking”.*

*- Male, Asian*

Another point made apparent was the lack of context for NICE guidelines for non-White patients/race, in addition to how the history of medicine has impacted BAME communities and their attitudes to healthcare. Moreover, some students felt that the compartmentalisation of teaching on clinical signs in non-White skin was unhelpful as it should be incorporated into the standard curriculum. This lack of diversity in the curriculum was corroborated by how both BAME and White students felt that their cultural competency regarding different ethnicities and religions was inadequate; many felt as though they were not prepared to encounter demographics outside of Wales, which are typically viewed as more diverse.

*“I think a token lecture isn’t helpful because you're encouraging people to compartmentalise race. If you say you're going to have this one lecture on race, then it's kind of like race applies to that one area of medicine, which is not right [...] So, if you're talking about a patient that is having food poisoning, you know somehow they have to be in Africa, whereas if you're talking about a patient that has squamous cell carcinoma it has to be a white Caucasian. We kind of stereotype people and then that kind of encourages us to miss diagnosis.”*

*- Female, Black*

*“I think for a lot of people they're already quite culturally competent, but I think some people aren't, and if the kind of experience they get from their safe university bubble is quite homogenous they're going to just take that with them into their workplace and onto placement and, unfortunately, like perpetuate this kind of cultural ignorance.”*

*- Male, White*

#### Support

Many participants, except for two, reported negative experiences when making complaints about race-based discrimination. Several students felt that their complaints had not been taken seriously upon initial reporting of the incidents, so had subsequently not made further complaints. Indeed, a few students, including those from a BAME background, mentioned how there were no actionable outcomes and a lack of response from student-colleagues and seniors, which not only deterred them from further reporting, but also left them feeling dismayed. Several BAME students found themselves in situations in which they felt uncomfortable to speak out due to hierarchical factors, the fear of being seen as ‘unprofessional’, and uncertainty in support from the medical school.

*“The support networks in place with specific reporting are just not there.. never in a million years, with the current support networks will I just ever report any cases of potential racism that comes towards me.”*

*- Male, Asian*

*“My experience in the medical school has been that flagging this sort of thing up doesn't get you anywhere [...] but this experience has made me think more ‘why is any NHS trust going to be different?’, because my Medical School doesn't think it's important to consider that I'm being discriminated against.”*

*- Female, Asian*

*“You get so used to complaining about these things, initially, and then you just stop because you don't feel listened to and then because you're so used to hearing it from everybody you just subconsciously start thinking it's normal.”*

*- Male, Asian*

## Discussion

### Statement of principal findings

In this qualitative study of students’ perspectives of the effects of ethnicity on experiences, most BAME students in the study group reported feeling disadvantaged during their studies, or unsure. Incidents were most reported during patient interactions, but a significant emphasis was highlighted through clinician interactions, as students felt that it led to a direct disadvantage to their education due to the influence clinicians had on learning and career opportunities. Disadvantages in education were most often linked to gender-based discrimination and this was reported by all BAME and non-BAME female participants. BAME participants also felt that to be offered opportunities, they had to be more forthright compared to their White counterparts. In the narratives by various BAME students, names that were deemed difficult to pronounce by patients and clinicians were a barrier in building rapport; this resulted in a distinct lack of engagement from patients and clinicians, or disrespectful and unprofessional comments. Identifying individuals by their name is the first step in cultivating both professional and personal relationships and would indeed be instrumental in improving learning experiences for BAME students, through asserting a mutual respect. Participants mentioned a lack of trust in their institution, procedures for escalating problems were unclear and students felt dismissed when escalating such issues. All participants identified microaggressions, whilst offensive comments and blatant racism were particularly emphasised by Black students.

Additionally, students felt that inappropriate and stigmatising language was used in teaching, which isolated BAME students. Students felt that the language used in the teaching curriculum could be outdated and did not prepare them for treating diverse populations.

### Comparison with literature

Current literature suggests that there are various reasons for the differential achievements of BAME students compared to their non-BAME counterparts, as indicated by the findings of this study. A focal point highlighted in these studies is how students’ relationships with university and clinical staff is a critical factor in their academic success. Two studies found that students specifically learnt the most when clinicians interacted with them [9, 10].

However, archaic language used in some lectures was reported as a barrier to the engagement of BAME students in comparison to non-BAME students, during lectures, and in clinical spaces. A similar study carried out in a West Midlands medical school demonstrated how students also felt that lecturers made culturally insensitive or inappropriate comments. Such racial microaggressions caused students to feel excluded and invalidated [11]. Although students in this study discussed patient experiences as a focal point for discriminatory events, other qualitative studies have not emphasised this, except for a 2019 study where patient experiences were perceived to impede learning [11].

The medical school has begun to take steps towards an improved awareness of cultural competency, as seen through their Mission Statement, instituting a review of the curriculum, and implementing Active Bystander Training for staff and students, including an Equality & Diversity agenda item into all committee and formal meeting agendas, and removing references deemed to be too ‘colonial’. Lack of cultural competency has been reported by the majority of students in this study. Studies have shown that staff, patients and students were all found to be limited in their cultural awareness [12, 13], highlighting the critical need for further education. Such a lack of cultural competency could potentially be rectified by greater representation and input from BAME clinical staff and lecturers, which the medical school has since been trying to implement. In this study, students felt that while the senior leadership team was diverse, there is a notable lack of Black staff members, which has been reported in two other studies [11, 13].

Moreover, lack of trust with institutions and lack of confidence in reporting procedures has been noted in other studies, whereby lack of guidance has been perceived as lack of care [11, 14]. For example, this present study found that student confidence was lost due to insufficient guidance on the protocols for reporting incidents of racism or unfair treatment.

Other studies identify the stereotype threat as a prominent theme, which affected students’ behaviour and caused them to resort to identity masking [10, 11, 13, 15]. BAME participants in this study felt that they were held to a higher professional standard, particularly Black participants, although this was not overtly linked to the anxiety of stereotyping.

Although name mispronunciation and avoidance were a prominent feature in this study, they are only mentioned briefly in two other studies [11, 15]. BAME-participants in this study highlighted how efforts to learn and pronounce conventionally ‘Non-Western’ names would have made them feel more integrated and valued within their team. This was exacerbated when clinicians would clearly pronounce Welsh names, which are similarly viewed as difficult to pronounce, however then go on to either avoid or make no effort to correctly pronounce BAME-student names.

Interactions with peers and academic support networks have been identified in other studies as barriers to success, in addition to contributing to the isolation felt by BAME students, however this was not mentioned in our study [10, 11, 13, 15].

### Strengths and weaknesses

This study had several important strengths and weaknesses. The one-to-one nature of the interviews promoted unrestricted conversation and allowed participants to narrate their personal experiences [16]. Whilst a single researcher of an ethnic minority background may have allowed some participants to feel comfortable sharing sensitive experiences, it may have conversely affected how White participants, or those from other ethnic groups, expressed their views.

Additionally, the small sample size may mean that the results from this study cannot be generalised and lack a larger scale representation. From a cohort of approximately 400 students invited, a response rate of 17 were able to interview. This may have been impacted by the time of year of interviews, as first and fourth year students were preparing for examination periods. Additionally, first- and second-year students had minimal clinical exposure at the point of interviews and may not have felt able to contribute to the survey. If more participants were included, different themes derived from the conversations may have emerged.

Qualitative research is susceptible to multiple types of bias. Moderator bias is difficult to avoid, but the interviewer tried to appear neutral and refrained from giving personal opinions. Also, being a peer to students on the course and of an ethnic-minority background, this would have impacted participant responses. However, the researcher’s age, sex and race were non-modifiable and may have affected participant responses [17]. Bias within questions was avoided by developing neutral, clear, and concise questions. Also, by allowing participants to answer ‘Unsure’, it allowed those without experience of a particular situation to admit this, thus removing unanswerable question bias. Another important type of bias is error; whilst impossible to remove, it can be reduced. The researcher encouraged all participants to take their time and accurately recall details of their experiences. Moreover, because results were analysed by the sole researcher, who is also an ethnic-minority student on the course, this would have introduced an element of reporting bias [17]. Ideally, there should have been triangulation of thematic analysis from another, objective researcher. To counteract reporting bias, a second researcher was asked to identify prominent themes. Furthermore, the results were compared with previous studies which meant that other perspectives were considered. Although invites were extended across all cohorts of the GEM course, it is likely that participants who responded are those with an interest in the topic and this introduces an element of participant bias. This type of bias is difficult to combat, as a limited number of students opted to participate, and to be selective would have further reduced the sample size.

### Implications and further studies

The findings from this study highlight the disadvantages that BAME students face on a UK medical course. Whilst the study comprised only a select cohort of students in Wales, the results align with findings from other studies across the UK [9–15]. However, since the study was undertaken, the medical school has taken several positive steps in the right direction; ‘decolonisation’ of the medical curriculum, equality and diversity training for faculty members and students alike, and strong BAME representation at all levels, including the senior management team, should be acknowledged. The senior management team have recognised the crucial need to listen to student reflections providing an insight into their experiences at medical school, which they have admitted, at times, were not easy to read. Moreover, a student led BAME medical society has created a cultural competency course and has worked with local GPs and students to improve their cultural awareness. Whilst these are important steps, it is imperative that further action oversees greater improvement for our BAME medical students, led by medical school senior management, and at a health board and Trust level.

An expansion of this study to other UK medical schools would be beneficial as medical schools in other regions have varying BAME demographics and differing cultures. There are several strengths of this study that should be replicated but a study of similar design should attempt to eliminate biases. Future researchers could invite a high number of students to participate which would hopefully increase the response rate. Furthermore, comparison of student experiences in medical schools with differing intakes of BAME students may be revealing - it would be interesting to see whether cohort size alone has an impact for BAME student experiences. Other strategies to reduce biases may include employing an independent interviewer who is not a participant on the course and another independent researcher to collate themes.

## Conclusion

This study aimed to explore students' experiences in GEM and how their experiences are affected by ethnicity. Students from this study identified clinician biases leading to less engagement with BAME students, and an unwillingness to learn their names. They also described challenges in building rapport with some patients and were more likely to experience overt racism. All students expressed instances of disappointment in language used in teaching and felt unsupported when reporting incidents. Stigmatisation of non-Western cultures was also noticeable in the university curriculum, creating a distinct disparity between cultures typically accepted as the ‘norm’, and those which are less familiar. Protected characteristics such as sex, disability, and religion/belief were also found to disadvantage some students. These findings are consistent with similar studies in other UK medical schools. And ultimately, this study may pave the way for future research into how the UK medical programme can continue to address these important issues. However, the university must enact significant change and take tangible steps towards their mission statement of “producing excellent, caring and inclusive clinicians, for a global society”.

## Supplementary Information

**Additional file 1. **Questions for interview.

## Data Availability

The datasets generated and/or analysed during the current study are not publicly available due participant confidentiality but are available from the corresponding author on reasonable request.
