# Supplementary material for: Students perspectives of the effect of ethnicity on experiences in a graduate entry medicine course in Wales: a qualitative study
Source: BMC Med Educ. 2026 Feb 17;26:475. doi: 10.1186/s12909-023-04852-7 (PMC13015125; doi:10.1186/s12909-023-04852-7)
Supplement: Supplementary file 1 — Additional file 1. Questions for interview. [file 12909_2023_4852_MOESM1_ESM.docx]

# Additional file 1 - Questions for Interview

BAME Student Experiences in Clinical Settings

- Gender
- Ethnicity
- What are some positive experiences that you can highlight during placements and why?
- What are some negative experiences that you can highlight during placements and why?
- During your time studying medicine, have you ever felt like you have been at a disadvantage or given less opportunities?
  - What do you believe are the factors that led you to be treated this way?
- Do you think race/ethnicity play a factor in how others are treated in the workplace, or yourself if you feel it applies?
  - In teaching
  - By patients
  - By doctors
  - By nurses
  - By other healthcare professionals
- How do you think the way things are taught could be improved?
  - In preparation for clinical teaching
  - During clinical teaching
